# The CXCL12/CXCR4 Signaling Axis Retains Neutrophils at Inflammatory Sites in Zebrafish

**DOI:** 10.3389/fimmu.2019.01784

**Published:** 2019-07-31

**Authors:** Hannah M. Isles, Kimberly D. Herman, Anne L. Robertson, Catherine A. Loynes, Lynne R. Prince, Philip M. Elks, Stephen A. Renshaw

**Affiliations:** ^1^Renshaw Laboratory, Department of Infection, Immunity, and Cardiovascular Disease, The Bateson Centre, University of Sheffield, Sheffield, United Kingdom; ^2^Elks Laboratory, Department of Infection, Immunity, and Cardiovascular Disease, The Bateson Centre, University of Sheffield, Sheffield, United Kingdom; ^3^Prince Laboratory, Department of Infection, Immunity, and Cardiovascular Disease, University of Sheffield, Sheffield, United Kingdom; ^4^Zon Laboratory, Division of Hematology/Oncology, Boston Children's Hospital and Harvard Medical School, Boston, MA, United States

**Keywords:** neutrophil, inflammation, chemokine, retention, CXCR4, CXCL12, zebrafish

## Abstract

The inappropriate retention of neutrophils at inflammatory sites is a major driver of the excessive tissue damage characteristic of respiratory inflammatory diseases including COPD, ARDS, and cystic fibrosis. The molecular programmes which orchestrate neutrophil recruitment to inflammatory sites through chemotactic guidance have been well-studied. However, how neutrophil sensitivity to these cues is modulated during inflammation resolution is not understood. The identification of neutrophil reverse migration as a mechanism of inflammation resolution and the ability to modulate this therapeutically has identified a new target to treat inflammatory disease. Here we investigate the role of the CXCL12/CXCR4 signaling axis in modulating neutrophil retention at inflammatory sites. We used an *in vivo* tissue injury model to study neutrophilic inflammation using transgenic zebrafish larvae. Expression of *cxcl12a* and *cxcr4b* during the tissue damage response was assessed using *in situ* hybridization and analysis of RNA sequencing data. CRISPR/Cas9 was used to knockdown *cxcl12a* and *cxcr4b* in zebrafish larvae. The CXCR4 antagonist AMD3100 was used to block the Cxcl12/Cxcr4 signaling axis pharmacologically. We identified that *cxcr4b* and *cxcl12a* are expressed at the wound site in zebrafish larvae during the inflammatory response. Following tail-fin transection, removal of neutrophils from inflammatory sites is significantly increased in *cxcr4b* and *cxcl12a* CRISPR knockdown larvae. Pharmacological inhibition of the Cxcl12/Cxcr4 signaling axis accelerated resolution of the neutrophil component of inflammation, an effect caused by an increase in neutrophil reverse migration. The findings of this study suggest that CXCR4/CXCL12 signaling may play an important role in neutrophil retention at inflammatory sites, identifying a potential new target for the therapeutic removal of neutrophils from the lung in chronic inflammatory disease.

## Introduction

The inappropriate retention of activated innate inflammatory cells at inflammatory sites is major driver of chronic inflammatory diseases including asthma, COPD and rheumatoid arthritis (1). Neutrophils are one of the first cell types recruited to sites of inflammation, where they are potent anti-microbial effectors through the phagocytosis of foreign material, generation of reactive oxygen species and the production of extracellular traps (2–4). These non-specific anti-microbial mechanisms promote a tissue microenvironment which is unfavorable to pathogens, but at the expense of host tissue integrity (5). Neutrophil removal from inflammatory sites is therefore tightly regulated to minimize collateral tissue damage, thereby preventing chronic inflammatory disease (6). Despite the global burden of chronic inflammatory diseases, there are currently no effective therapies to treat the neutrophilic component of these conditions, highlighting a need to identify novel drug targets to promote the successful resolution of inflammation.

It has been known for 30 years that neutrophils undergo apoptosis followed by efferocytosis by macrophages, and this is the best characterized mechanism by which neutrophils are removed from inflammatory sites (7, 8). Although methods to both accelerate and delay apoptosis have been identified (9–13), none of these are yet in clinical use for inflammatory disease. More recently, reverse migration has been identified as a mechanism by which neutrophils redistribute into the tissue or vasculature surrounding the inflammatory site, an anti-inflammatory mechanism which is thought to disperse the inflammatory burden (10, 11, 14, 15). Reverse neutrophil migration has been visualized *in vivo* using transparent zebrafish larvae (10, 16–18), as well as in mice (14) and human neutrophils (19). *In vitro* studies using microfluidics devices identified that over 90% of human neutrophils can reverse their directionality away from a chemoattractant over distances of 1,000 μm (20). The mechanisms governing this newer phenomenon are not fully understood, though it is clear that the capacity of neutrophils to cause host tissue damage is increased when either apoptosis or reverse migration are impaired, resulting in the inappropriate retention of neutrophils at the inflammatory site (21). Understanding neutrophil reverse migration represents novel therapeutic avenues to treat neutrophil mediated chronic inflammation.

During inflammation, neutrophils respond to complex guidance cues provided in part by chemokine gradients which promote the directed migration of neutrophils from the circulation and into inflamed tissues (22). More recently, a role for chemokine signaling in modulating neutrophil reverse migration has been identified (15, 18), making chemokine receptors an attractive target for investigation. Computational modeling and *in vivo* studies of reverse migration have shown that this process likely occurs as a result of the stochastic redistribution of neutrophils following their desensitization to local chemotactic gradients over time (11, 16, 18). In zebrafish, neutrophil reverse migration can be delayed by stabilizing HIF1α which promotes neutrophil retention at inflammatory sites (10), suggesting that downstream HIF signaling targets retain neutrophils at inflammatory sites. Work by our group and others has shown that this retention of neutrophils at inflammatory sites is both mechanistically important (10, 21), and can be manipulated therapeutically (11, 13, 18), yet the molecular mechanisms remain to be elucidated.

CXCR4 is a G protein coupled receptor expressed by many leukocytes, which exerts its biological functions by signaling through its major ligand CXCL12 (formerly known as stromal derived factor 1). CXCL12/CXCR4 signaling is a key retention signal for neutrophil release into the blood circulation from hematopoietic tissues, the crucial role of which is highlighted in patients with warts, hypogammaglobulinaemia, infection, and myelokathesis (WHIM) syndrome. Gain of function WHIM mutations result in increased CXCR4 signaling, the consequence of which is severe neutropenia with increased neutrophil retention in the bone marrow (23).

There is growing evidence to support a role for CXCL12/CXCR4 in neutrophil retention in the context of inflammatory disease. Tissue infiltrated neutrophils from patients with chronic inflammatory lung diseases and rheumatoid arthritis have increased CXCR4 surface expression (24). Neutrophil surface expression of CXCR4 is increased after extravasation into injured lungs in mice (25) and in human tissue samples, where pulmonary CXCL12 expression increases during acute lung injury (26). Additionally, the inhibition of CXCL12 using blocking antibodies prevented the accumulation of neutrophils in the lung during the late stages of LPS induced lung injury (25). Based on this evidence we hypothesized that CXCL12/CXCR4 functions as a retention signal in the context of tissue damage, functioning to maintain active neutrophils at the inflammatory site.

Here we present a new role for the CXCL12/CXCR4 signaling axis in the retention of neutrophils at inflammatory sites and demonstrate a role for neutrophil retention signaling in modulating inflammation resolution in zebrafish larvae. Using both pharmacological and genetic approaches to manipulate the CXCL12/CXCR4 signaling axis, we demonstrate that interruption of CXCR4 signaling increases neutrophil reverse migration. We have identified a druggable signaling axis which could be a therapeutic target to remove excessively retained neutrophils at inflammatory sites during disease.

## Materials and Methods

### Zebrafish Husbandry and Ethics

To study neutrophils during inflammation *TgBAC(mpx:EGFP)i114* (known as mpx:GFP) (27) zebrafish larvae were in-crossed. To study gene expression by whole mount *in situ* hybridization, wildtype pigment-less *nacre* (28) larvae were in-crossed. For reverse migration assays, *Tg(mpx:GAL4.vp16)sh267;Tg(UAS:Kaede)i222* (known as mpx:kaede) were in-crossed.

All zebrafish were raised in the Bateson Centre at the University of Sheffield in UK Home Office approved aquaria and maintained following standard protocols (29). Tanks were maintained at 28°C with a continuous re-circulating water supply and a daily light/dark cycle of 14/10 h. All procedures were performed on embryos <5.2 dpf which were therefore outside of the Animals (Scientific Procedures) Act, to standards set by the UK Home Office.

### Neutrophil Specific Expression of Zebrafish Genes

Gene expression was assessed using an RNA sequencing database from FACS sorted GFP positive cells from 5 dpf zebrafish and FPKM values for genes of interest were extracted (30) (data deposited on GEO under accession number GSE78954). For single cell analysis, gene expression values were extracted from the BASiCz (Blood atlas of single cells in zebrafish) cloud repository (31). Cells of the neutrophil lineage were analyzed for expression of *cxcr4a, cxcr4b, cxcl12a*, and *cxcl12b*.

### WISH Probe Synthesis

The WISH antisense RNA probe for *cxcl12a* was synthesized from linearised plasmid DNA obtained from a plasmid vector containing the zebrafish *cxcl12a* coding sequence. Following transformation and DNA purification, the plasmid was linearised by restriction digest using EcoR1 [New England Biolabs (NEB), Herts, UK]. The RNA probe was transcribed from linearised DNA using an SP6 RNA digoxigen labeling kit (Roche). One μg of linearised DNA was incubated in a final volume of 20 μl containing transcription reagents and transcription reaction was performed according to standard protocols (Roche).

### Whole Mount *in situ* Hybridization

Nacre larvae were anesthetized in tricaine following tail fin transection at time points indicated in the figure legends alongside uninjured, age-matched controls. No more than 20 larvae were transferred to 1 ml Eppendorf tubes and excess liquid was removed without damaging larvae. One ml of paraformaldehyde (PFA) at 4°C was added to Eppendorf tubes for the fixation step, and left overnight at 4°C. Larvae were washed and transferred into 100% methanol and stored at −20°C for at least 24 h prior to use. WISH was performed using standard protocols (32) using an antisense DIG labeled probe for zebrafish *cxcl12a*.

### CRISPR/Cas9 Reagents

Synthetic SygRNA® consisting of crRNA and tracrRNA (Merck) in combination with Cas9 nuclease protein (Merck) was used for gene editing. Transactivating RNAs (tracrRNA) and gene specific CRISPR RNAs (crRNA) were resuspended to a concentration of 20 μM in nuclease free water containing 10 mM Tris-HCl pH8. SygRNA® complexes were assembled on ice immediately before injection using a 1:1:1 ratio of crRNA:tracrRNA:Cas9 protein. Gene-specific crRNAs to target *cxcr4b* and *cxcl12a* were designed using the online tool CHOPCHOP (http://chopchop.cbu.uib.no/). We used the following crRNA sequences, where the PAM site is indicated in brackets: ***cxcr4b:*** CAGCTCTGACTCCGGTTCTG(GGG) ***cxcl12a:***CTCTACCAGGCTGATGGGCT(TGG).

### Microinjection of SygRNA® Into Embryos

A 1 nl drop of SygRNA®:Cas9 protein complex was injected into mpx:GFP embryos at the one-cell stage. Embryos were collected at the one cell stage and injected using non-filament glass capillary needles [Kwik-Fil™ Borosilicate Glass Capillaries, World Precision Instruments (WPI), Herts, UK]. RNA was prepared in sterile Eppendorf tubes. A graticule was used to measure 0.5 nl droplet sizes to allow for consistency of injections. Injections were performed under a dissecting microscope attached to a microinjection rig (WPI) and a final volume of 1 nl was injected.

### Genotyping of Crispant Larvae

To determine the efficiency of CRISPR/Cas9 to induce site-specific mutations in injected larvae, we used restriction digest assays (Supplemental Figure 2). CRISPR guides were designed to target sequences containing restriction digest sites, such that when indels were introduced by DNA repair, the restriction site is disrupted. Genomic DNA was extracted from individual larvae at 2 dpf. Larvae were placed individually in 0.2 ml PCR tubes in 90 μl 50 mM NaOH and boiled at 95° for 20 min. Ten μl Tris-HCl pH 8 was added as a reaction buffer and mixed thoroughly. RT-PCR using Firepol® (Solis BioDyne) was used to amplify a 235 bp region (for *cxcr4b*) and a 259 bp region (for *cxcl12a*) around the PAM site. Gene specific primers were designed using the Primer 3 web tool (http://primer3.ut.ee/). Primer sequences were as follows: ***cxcrb4_fw****TCCCGTATACTGTAGGGAGGA* ***cxcr4b_rev*** *TTTTTGCATTTTGTTTTCTTG* ***cxcl12a_fw****TTCTCTGTGGGACTGTGTTGAC* ***cxcl12a_rev*** *TTCGAAAATTTGACCCAAAAGT*. Restriction enzyme digests were then performed using bsII at 55° for 2 h (for *cxcr4b*) and bstXi (New England Biolabs) at 37° for 2 h (for *cxcl12a*). Products were run using gel electrophoresis on a 2% gel (Supplemental Figure 2).

### Inflammation Assays in Crispant Larvae

To induce an inflammatory response, chorions of zebrafish larvae at 2 dpf were removed using sterile laboratory tweezers and larvae were anesthetized in Tricaine (0.168 mg/ml; Sigma-Aldrich) in E3 media and visualized under a dissecting microscope. Tail-fins were transected consistently using a scalpel blade (5 mm depth, WPI) by slicing immediately posterior to the circulatory loop, ensuring the circulatory loop remained intact as previously described (27). Larvae were maintained at 28°C in fresh E3 media in a 24 well plate. Neutrophils at the wound site were counted at timepoints indicated in figure legends using a fluorescence stereo microscope.

### Compound Treatment of Larvae for Inflammation Resolution Assays

To study the resolution of inflammation, neutrophils were counted at the wound site at intervals during the resolution phase from 8 to 24 h post injury in 2 dpf mpx:GFP larvae, as indicated in figure legends. Larvae were dechorionated and anesthetized prior to injury by tail-fin transection and left to recover at 28°C in fresh E3 media in petri dishes (60 larvae per plate). Larvae were screened for good neutrophil recruitment (around 20 neutrophils at the wound site) at 3.5 hpi. AMD3100 (Sigma-aldrich) was administered to larvae at 4 hpi through injection into the duct of Cuvier at a final concentration of 20 μM. AMD3100 was always tested alongside the appropriate vehicle control. Neutrophils at the wound site were counted at 6 hpi at the peak of recruitment, and at 8 hpi for inflammation resolution using a fluorescence stereo microscope (Leica).

### Percentage Resolution Calculations

To determine resolution of the neutrophil component of inflammation, experiments were performed with larvae maintained separately in a 96 well plate to follow individual larvae over time. Percentage reduction in neutrophil counts at the wound was calculated as **[**(Neutrophil counts at peak recruitment – neutrophil counts at 8hpi)/neutrophil counts at peak recruitment]^*^100.

### Whole Body Neutrophil Counts

Whole body neutrophil counts were measured in mpx:GFP larvae at time points indicated in figure legends. Larvae were mounted in 1% agarose with tricaine and a single slice image was taken using a 4x NA objective lens on an Eclipse TE2000 U inverted compound fluorescence microscope (Nikon UK Ltd., Kingston upon Thames, UK). A GFP-filter was used at excitation of 488 nm. Two images were taken per larvae, one of the head region and one of the tail region. Neutrophils were counted manually from both images and combined to give a whole body neutrophil count.

### Reverse Migration Assay

Reverse migration assays were performed using larvae expressing the photoconvertible protein kaede under the neutrophil specific mpx promoter: *TgBAC(mpx:GAL4-VP16); Tg(UAS:Kaede)i222*. At 3 dpf larvae were anesthetized and injured by tail-fin transection and left to recover at 28°C. Larvae were screened for good neutrophil recruitment at 4 hpi. AMD3100 was administered by incubation in low melting point agarose containing tricaine at 5 hpi in 3 dpf larvae. Photoconverstion of kaede labeled neutrophils at the wound site was performed using an UltraVIEWPhotoKinesis™ device (Perkin Elmer and Analytical Sciences) on an UltraVIEWVoX spinning disc confocal laser imaging system (Perkin Elmer). The photokinesis device was calibrated using a coverslip covered in photobleachable substrate (Stabilo Boss™, Berks UK). Photoconverstion was perfomed using a 405 nm laser at 40% using 120 cycles, 250 pk cyles and 100 ms as previously published (10). Following calibration, a region of interest was drawn at the wound site between the edge of the circulatory loop and encapsulating the entirety of the wound edge. Successful photoconversion was detected through loss of emission detected following excitation at 488 nm, and gain of emission following 561 nm excitation. Larvae were then transferred to an Eclipse TE2000-U inverted compound fluorescence microscope with 10x NA objective lense to acquire images using an andor zyla 5 camera (Nikon). Time lapse imaging of neutrophil reverse migration was performed for 5 h using 2.5 min intervals using GFP and mCherry filters with 488 and 561 nm excitation, respectively. For quantification of reverse migration, NIS elements software was used to compress z-slices into maximum intensity projections. A region of interest was drawn around the region away from the wound site. For quantification of neutrophils moving away from the wound site, a binary threshold was applied to images to detect mCherry neutrophils from background noise and NIS elements software calculated the number of objects detected in the ROI at each time point, providing a read out of reverse migration.

## Results

### *cxcr4b* and *cxcl12a* Are Expressed Following Tissue Damage in Zebrafish

**Figure 1:**
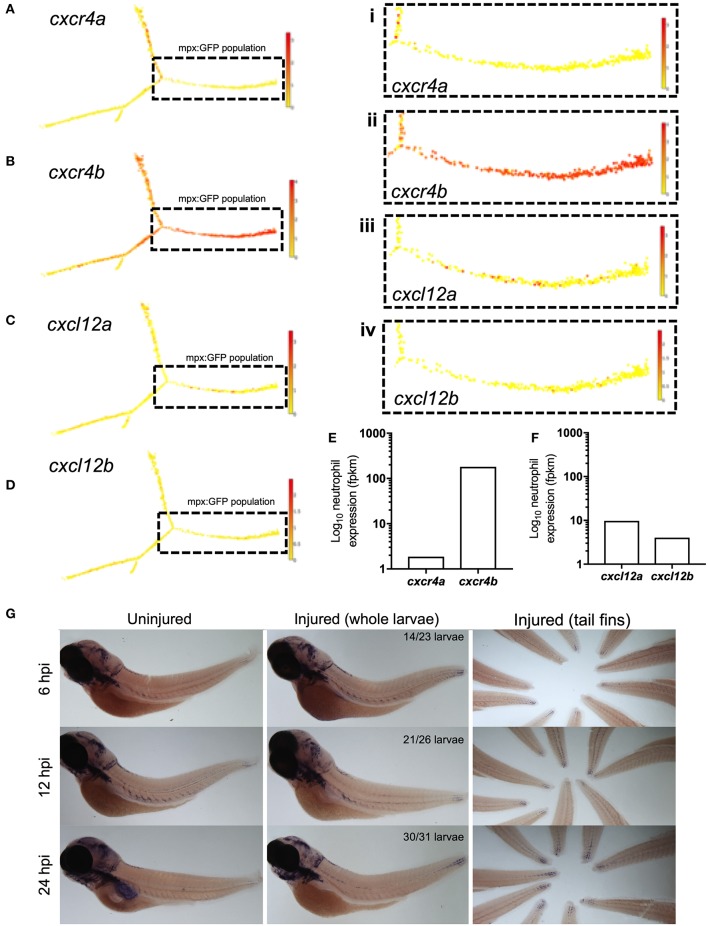
*cxcr4b* and *cxcl12a* are expressed following tissue damage in zebrafish. **(A–D)** Single-cell gene expression profiles for *cxcr4* and *cxcl12* in the zebrafish blood lineage. Single cell gene expression values extracted from the Sanger BASiCz zebrafish blood atlas. Circles represent individual cells color coded where red is high expression and yellow is no expression. Neutrophil lineage (mpx:GFP positive) is highlighted by black dashed box and expanded in (i–iv). **(E,F)** RNA sequencing of FACS sorted GFP positive cells from *TgBAC(mpx:GFP)i114* zebrafish larvae at 5 days post fertilization. FPKM values illustrate neutrophil expression of **(E)** *cxcr4a* and *cxcr4b* and **(F)** *cxcl12a* and *cxcl12b*. **(G)** Whole mount *in situ* hybridization using an antisense DIG labeled RNA probe for *cxcl12a* mRNA. Wildtype *nacre* zebrafish larvae were injured and fixed in PFA at 6, 12, and 24 h post injury, along with uninjured age-matched control larvae. Left and middle panels show whole zebrafish larvae at timepoints indicated, right panel shows tail fins of a representative experiment. Quantification shows number of larvae which look like representative image from 2 independent experiments.

Zebrafish have two paralogues for CXCR4 and CXCL12, following a genome duplication event in teleost evolution. The expression of *cxcr4a* and *cxcr4b* is mutually exclusive in most cell lineages, indicating partitioned ancestral functions in different tissues (33). In zebrafish larvae Cxcl12a is produced in regions of neutrophil development alongside expression in the head, pronephric duct and CHT of zebrafish larvae at 2dpf (34), as well as in the regenerating fins of adult zebrafish (35, 36). To determine the gene expression of Cxcr4 and Cxcl12 during the cellular response to tissue damage in zebrafish larvae, we first investigated neutrophil expression of *cxcr4 and cxcl12*. We studied published datasets combining RNA sequencing of mpx:GFP positive zebrafish larval neutrophils and single-cell RNA sequencing data from adult zebrafish blood lineages (30, 31). In adult zebrafish neutrophils, *cxcr4b* is highly expressed by the neutrophil lineage whilst *cxcr4a* is undetectable (Figures 1A,B). *Cxcl12a* is expressed by a small population of adult zebrafish neutrophils, albeit far fewer than *cxcr4b*, whilst *cxcl12b* is expressed by very few cells (Figures 1C,D). We analyzed larval stage neutrophil RNA sequencing data (30), and found that fragments per kilobase million (fpkm) values for *cxcr4b* were over 100-fold higher than the fpkm values for *cxcr4a* (Figure 1E), confirming that *cxcr4b* is the predominantly expressed isoform in larval zebrafish neutrophils. Furthermore, we confirmed that expression of *cxcl12a* and *cxcl12b* was low in larval neutrophils (Figure 1F).

Zebrafish Cxcr4b is activated by the chemokine Cxcl12a (37), we therefore investigated the expression of *cxcl12a* during the inflammatory response in 3dpf larvae. To induce an inflammatory response we used our well-characterized tail-fin injury model of spontaneously-resolving neutrophilic inflammation (27), where neutrophil recruitment is observed between 0 and 6 h post injury (hpi) and resolution of the neutrophilic component of inflammation occurs between 6 and 24 hpi. Whole mount *in situ* hybridization was used to detect *cxcl12a* mRNA at the wound site in 3 dpf larvae following tail fin transection. *Cxcl12a* mRNA expression was detected in injured larvae as early as 6 hpi during the recruitment phase (Figure 1G). Interestingly, *cxcl12a* mRNA expression continued to increase throughout the resolution phase up to 24 hpi (Figure 1G) in keeping with other reports of *cxcl12* expression following fin injury. These findings show the expression of *cxcr4b* by neutrophils and *cxcl12a* at the tissue injury site during the inflammatory response in zebrafish.

### Genetic Manipulation of the CXCL12/CXCR4 Signaling Axis Accelerates Inflammation Resolution

**Figure 2:**
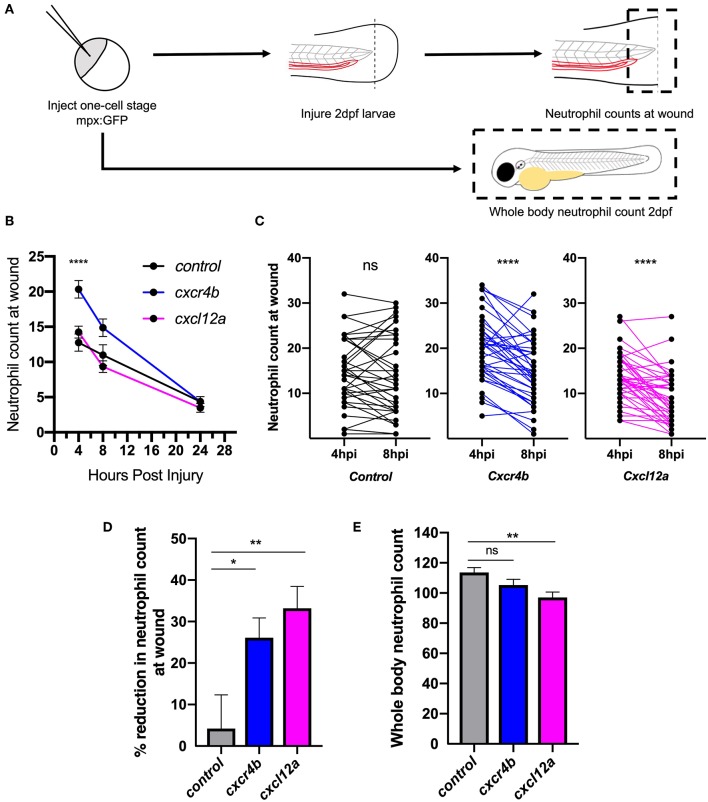
Knockdown of *cxcr4b* using CRISPR/Cas9 accelerates inflammation resolution. **(A)** Experimental schematic of CRISPR/Cas9 experiments in 2dpf mpx:GFP larvae. **(B–D)** CRISPR/Cas9-mediated knockdown of *cxcr4b* and *cxcl12a* accelerates inflammation resolution. **(B)** Time course of neutrophils at the wound site in control *tyr* crispant larvae (black line), *cxcr4b* crispant larvae (blue line), and *cxcl12a* crispant larvae (pink line) at 4, 8, and 24 hpi. Error bars shown are mean ± SEM. Groups were analyzed using an ordinary one-way ANOVA and adjusted using Tukey's multi comparison test. ^****^p < 0.001 *n* = 36 from 3 independent experiments. **(C)** Neutrophils at the wound site in individual larvae (black dots) at 4 and 8 h post injury in *tyr* crispant larvae (black), *cxcr4b* crispant larvae (blue), and *cxcl12a* crispant larvae (pink). Groups were analyzed using a paired t-test. ^****^p < 0.001 *n* = 36 from 3 independent experiments. **(D)** Resolution of the neutrophil component of inflammation was calculated between 4 and 8 hpi. Groups were analyzed using an ordinary one-way ANOVA and adjusted using Tukey's multi comparison test. ^*^p < 0.04, ^**^p < 0.004. **(E)** Whole body neutrophil numbers were measured in mpx:GFP crispant larvae at 2 dpf. *n* = 30–35 per group from 3 independent experiments. Error bars shown are mean ± SEM. Groups were analyzed using an ordinary one-way ANOVA and adjusted using Tukey's multi comparison test, ^**^p < 0.005.

After determining that *cxcl12a* was expressed at the wound site in injured larvae, we next investigated neutrophil responses to tissue injury in the absence of the CXCL12/CXCR4 signaling axis. We hypothesized that if CXCL12/CXCR4 signaling was a neutrophil retention signal, inhibition of this pathway would accelerate neutrophil removal from the tissue injury site. We used CRISPR/Cas9 to study the role of Cxcl12a and Cxcr4b in neutrophilic inflammation resolution using the *TgBAC(mpx:GFP)i114* transgenic reporter line (27). A crRNA targeting the pigment gene tyrosinase (*tyr*) (38) was used for control injections and to allow for visual identification of successful knockdown. Knockdown of *tyr* produces an albino phenotype in zebrafish larvae (Supplemental Figures 1A,B) without affecting neutrophil development or the neutrophilic inflammatory response (Supplemental Figures 1C,D). We generated *cxcr4b* or *cxcl12a* “crispants” (newly generated “F0” CRISPR/Cas9-mediated mutants) and transected tail-fins at 2 dpf, counting neutrophils at the wound site at 4, 8, and 24 hpi (Figures 2A,B). Neutrophil counts in *cxcr4b* crispants were significantly increased at the wound site during the neutrophil recruitment phase (4 hpi), consistent with enhanced release of *cxcr4b* mutant neutrophils from their site of production (39) (Figure 2B). *Cxcl12a* crispants showed no difference in neutrophil recruitment (Figure 2B). No significant difference in neutrophil numbers at the wound site was detected between groups at 8 and 24 hpi (Figure 2B). We assessed the difference in neutrophil counts at the wound site between 4 and 8 hpi in each group. No significant difference in neutrophil number at the wound site was detected in control larvae, whilst both *cxcr4b* and *cxcl12a* crispants exhibited a significant decrease in neutrophil number (Figure 2C), suggesting neutrophil removal from the wound site in increased compared to control. To control for the increase in early neutrophil recruitment measured in Cxcr4b crispants, we calculated percentage reduction in neutrophil counts at the wound in individual larvae between 4 and 8 hpi. Both Cxcr4b and Cxcl12a crispants had significantly higher percentage reduction in wound neutrophils compared to control larvae (Figure 2D). Whole body neutrophil numbers were not affected in *cxcr4b* crispants, but were significantly reduced in *cxcl12a* crispants (Figure 2E). These data demonstrate that loss of Cxcl12/Cxcr4 signaling accelerates resolution of the neutrophilic component of inflammation in zebrafish larvae, suggesting that the CXCL12/CXCR4 signaling axis is required for neutrophil retention at inflammatory sites.

### Pharmacological Inhibition of CXCR4 Accelerates Inflammation Resolution

**Figure 3:**
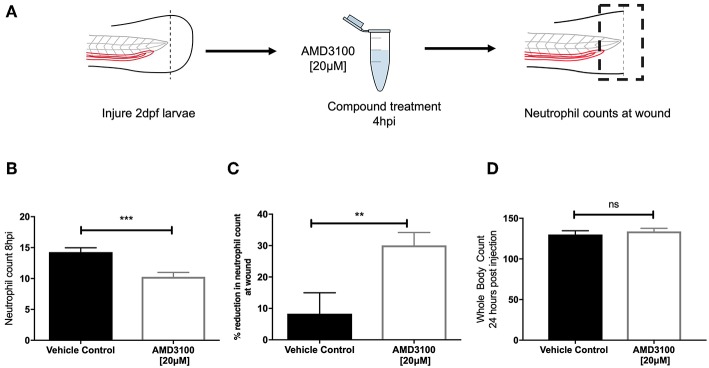
Inhibition of CXCR4 using AMD3100 accelerates inflammation resolution. **(A)** Experimental schematic of inflammation resolution experiments with AMD3100 compound treatment. **(B)** Number of neutrophils at the wound site in injured 2 dpf mpx:GFP larvae treated with AMD3100 or vehicle control at 8 hpi. Groups were analyzed using an unpaired *t*-test, ^***^p < 0.0002, *n* = 55 larvae from 5 independent experiments. **(C)** Resolution of the neutrophil component of inflammation for larvae treated with vehicle control or AMD3100. Groups were analyzed using an unpaired *t*-test, ^**^p < 0.008 *n* = 32 larvae from 3 independent experiments. **(D)** Whole body neutrophil counts in 3 dpf mpx:GFP larvae 24 h post administration of AMD3100 or vehicle control. Groups were analyzed using an unpaired *t*-test, *n* = 26 larvae from 3 independent experiments.

Genetic knockdown of CXCR4 signaling causes neutrophil release from the caudal haematopoietic tissue (CHT), enhancing neutrophil recruitment, confounding assessment of inflammation resolution. To circumvent this, we used the CXCR4 antagonist AMD3100 to block CXCR4 signaling in a time-sensitive fashion (Figure 3A). At 8 hpi a significant decrease in neutrophil counts at the wound site was detected in AMD3100 treated larvae (Figure 3B). Percentage inflammation resolution was significantly higher in AMD3100 treated larvae (Figure 3C), whilst whole body neutrophil counts were not affected by AMD3100 at 24 h post administration (Figure 3D). Together these data demonstrate that pharmacological inhibition of CXCR4 in larvae which have mounted a normal response accelerates resolution of the neutrophilic component of inflammation, further supporting a role for CXCL12/CXCR4 signaling in neutrophil retention signaling at sites of tissue damage.

### Inhibition of CXCL12/CXCR4 Signaling Increases Neutrophil Reverse Migration

**Figure 4:**
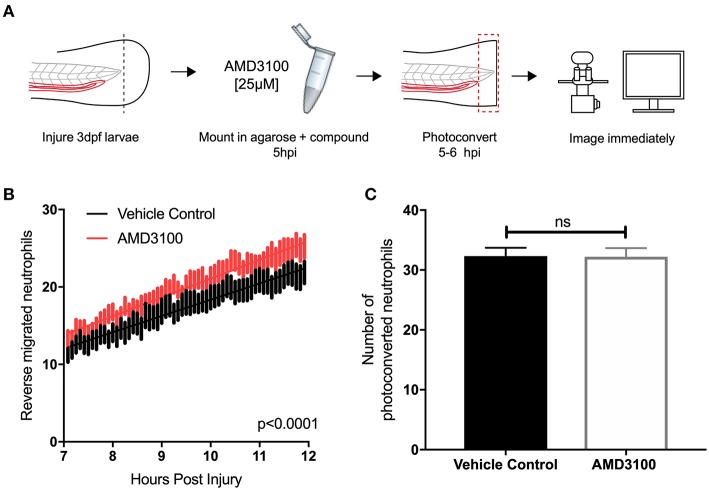
Inhibition of CXCR4 using AMD3100 accelerates neutrophil reverse migration. **(A)** Experimental schematic of neutrophil reverse migration assay. Tail fin transection was performed on 3 dpf mpx:kaede larvae. Larvae were mounted in a 1% agarose solution containing AMD3100 or vehicle control at 5 hpi. Neutrophils at the wound site were photoconverted at 5 hpi from green to red fluorescence. Time lapse imaging was performed from 7 to 12hpi. **(B)** The number of neutrophils which moved away from the wound site into a defined region of interest was quantified from 7 to 12 h post injury in larvae treated with a vehicle control (black) or AMD3100 (red). Error bars shown are SEM, line of best fit shown is calculated by linear regression. *P*-value shown is for the difference between the two slopes *p* < 0.0001, *n* = 35 larvae from 6 independent experiments. **(C)** Number of neutrophils photoconverted between 5 and 6 h post injury in larvae treated with vehicle control or AMD3100. Data shown are mean ± SEM, groups were analyzed using an unpaired *t*-test.

Two principal mechanisms of inflammation resolution have been described: neutrophil apoptosis followed by efferocytosis by macrophages, and reverse migration of neutrophils away from inflammatory sites. In zebrafish larvae, neutrophil reverse migration is the predominant mode of inflammation resolution (10, 11, 40). We have previously proposed that neutrophil release from inflammatory sites is best explained by the desensitization of neutrophils to local chemokine gradients (16). This led us to the specific hypothesis that inhibition of CXCL12/CXCR4 signaling would accelerate reverse migration by accelerating neutrophil desensitization to CXCL12 gradients. To study neutrophil reverse migration, we used a well-described photoconversion approach to study the reverse migration of neutrophils from a wound site (10, 11, 13, 17). AMD3100 was administered to *TgBAC(mpx:GAL4-VP16); Tg(UAS:Kaede)i222* (referred to as mpx:kaede) larvae at 5 hpi and neutrophils at the wound site were photoconverted and tracked during the resolution phase (Figure 4A). Neutrophil migration away from the wound site was significantly higher in larvae treated with AMD3100 (Figure 4B), an effect which was not due to a difference in the number of photoconverted neutrophils (Figure 4C). Together these data demonstrate that inhibition of CXCL12/CXCR4 signaling can increase inflammation resolution by accelerating neutrophil reverse migration, identifying this signaling axis as a potential therapeutic target to specifically remove inflammatory neutrophils without affecting the normal recruitment of neutrophils to new inflammatory or infectious lesions.

## Discussion

A large body of evidence now exists to suggest a role for the CXCL12/CXCR4 signaling axis in modulating neutrophil behavior in chronic inflammatory disease. Aside from generation of neutrophil retention signals in multiple physiological settings (41, 42), neutrophils taken from patients with chronic inflammatory disease have increased CXCR4 expression, and CXCL12 is produced at sites of injury, including the lung (24, 25). A specific role for the CXCL12/CXCR4 signaling axis in retaining neutrophils in the CHT has recently been suggested following the study of neutrophil behavior in zebrafish Cxcr4b and Cxcl12a mutant larvae (39). Our study provides evidence that the CXCL12/CXCR4 signaling axis is important in modulating neutrophil migration away from sites of inflammation, identifying a potential new therapeutic target for chronic inflammatory disease.

Computational modeling of reverse migration previously performed by our group demonstrated that neutrophil reverse migration is best described as a process of stochastic redistribution of neutrophils back into the tissue rather than their active migration away from the wound site (16). These data further support our suggestion that neutrophil reverse migration is initiated following desensitization to chemokine gradients at the wound site rather their active migration away from chemorepulsive gradients (fugetaxis). Cellular desensitization to external gradients is a characteristic feature of signaling through G protein coupled receptors, many of which are expressed on the surface of neutrophils (43). A retention signal generated through chemokine receptor signaling would require expression of the chemokine within the inflamed tissue and the receptor on the neutrophil surface. Our analysis of RNA sequencing from FACS sorted zebrafish larval neutrophils and adult single-cell RNA sequencing shows that at both larval and adult stages of development, the predominantly expressed isoform of CXCR4 in zebrafish neutrophils is *cxcr4b*, whilst *cxcr4a* was undetectable. This is in keeping with RT-PCR performed on FACS sorted larval zebrafish neutrophils (34). Interestingly, RT-PCR performed on adult zebrafish whole kidney marrow suggests that both *cxcr4b* and *cxcr4a* are expressed by neutrophils in the adult stage (34). Our analysis of single-cell RNA sequencing data provides a more sensitive assay to look at individual neutrophil RNA expression, therefore it is likely that zebrafish neutrophils do not express *cxcr4a* in adulthood. Furthermore, we demonstrate that mRNA for the major ligand for this receptor, Cxcl12a, is expressed at the wound site during inflammation. The *cxcl12a* expression pattern we observed in uninjured larvae was comparable to that observed by other groups earlier in zebrafish development at 2dpf (34). Expression of *cxcl12a* mRNA appeared to increase at the wound site throughout the time course of inflammation, in keeping with a significant body of evidence that illustrates a role for CXCL12 in tissue repair (35, 36, 44). It has been proposed that Cxcl12a is important in providing directional guidance cues to regulate endothelial cell migration during arterial morphogenesis in the regenerating fin (45). Expression of *cxcl12a* is detected by WISH in injured adult tail fins from 1 day post amputation and persists during fin regeneration until 5 days post amputation (35).

The role for the CXCL12/CXCR4 signaling axis in zebrafish developmental processes has been elucidated largely using genetic studies to knock down the genes encoding the CXCR4 and CXCL12 proteins (37, 46, 47). The high efficiency of somatic mutation by CRISPR/Cas9 in injected F0 animals yields up to 99% somatic mutagenesis and biallelic gene disruption, enabling direct phenotypic analysis without the requirement for raising stable F2 adults (38, 48, 49). When using CRISPR/Cas9 to disrupt *cxcr4b* and *cxcl12a*, we achieved genomic disruption by introducing INDELs in >90% injected F0 larvae (Supplemental Figure 2). In our studies, knockdown of *cxcr4b* increased neutrophil recruitment to the wound site in crispant larvae. C-terminal truncations of Cxcr4b specifically in neutrophils (such as those found in WHIM syndrome patients) prevents receptor internalization and increases sensitivity to Cxcl12a gradients, thus retaining them in the caudal hematopoietic tissue (CHT) inappropriately (34). Neutrophils in WHIM zebrafish larvae are unable to respond to wound-generated gradients effectively, hence neutrophil recruitment to inflammatory sites is reduced in these larvae (34). Conversely, in the Cxcr4b *odysseus* mutant where Cxcr4b signaling is impaired, the number of neutrophils available to be recruited to tissue damage is increased (39), thus our findings are in keeping with the F2 mutant phenotype (39). Neutrophil recruitment toward Cxcl12a was not increased in our experiments, although this could be attributed to Cxcl12a larvae displaying significantly reduced whole body neutrophil counts. Inflammation resolution was significantly increased in both Cxcr4b and Cxcl12a crispant larvae, suggesting that genetic manipulation of both genes results in the same effect in terms of inflammation resolution.

One of the advantages of using the zebrafish as a model to study inflammation is that chemical compounds can be used to manipulate signaling pathways, where several compounds which target neutrophils have been identified using this approach (9, 11, 12). AMD3100 is a non-peptide bicyclam which is able to specifically antagonize the CXCR4 receptor at three main interaction residues located around the main ligand binding pocket of CXCR4 in transmembrane domains IV, VI and VII. Binding of AMD3100 competitively inhibits binding of CXCL12 and prevents subsequent downstream signaling (50). AMD3100 has been used to inhibit the CXCL12/CXCR4 signaling axis in zebrafish larvae, where concentrations ranging from 10 to 30 μM have been administered to larvae through incubation in fish water for up to 24 h (51), a concentration range which we remained within for our own experiments. Our results from both genetic and pharmacological manipulation of Cxcr4b and Cxcl12a demonstrate that inhibition of CXCL12/CXCR4 signaling accelerates inflammation resolution. We propose that AMD3100 is able to accelerate inflammation and reverse migration by competitively binding the CXCR4 receptor and preventing signaling downstream, thus recapitulating what would happen at a higher concentration of Cxcl12a later in the inflammatory response. AMD3100 can also act as an allosteric agonist of CXCR7 (52), which functions as a decoy receptor for CXCL12, with a role in cell generation of self-gradients which is crucial for proper migration of primordial germ cells toward their targets in zebrafish (53). Activation of CXCR7 fails to couple to G-proteins and to induce chemokine receptor mediated cellular responses, so AMD3100 is unlikely to activate downstream signaling pathways (54). Cxcr7 may modulate neutrophil sensitivity to Cxcl12, through its scavenging of the chemokine which reduces the level of Cxcl12 in the local tissue environment (55). However, as zebrafish larval neutrophils do not express this receptor (30) (data not shown), it is unlikely that scavenging through Cxcr7 is involved.

Reverse migration is impaired in Cxcr2 deficient zebrafish larvae where neutrophils are inappropriately retained at the wound site (18). It has been proposed that altered susceptibility of neutrophils to gradients at the wound site in Cxcr2 deficient larvae drives their passive migration away from the wound site. Our data are compatible with these findings, as the CXCR4 and CXCR2 signaling axis is known to antagonistically regulate neutrophil retention in other models (42). It would be interesting to speculate that the combined outcome of signaling through both CXCR4 and CXCR2 could modulate the reverse migration of neutrophils during inflammation resolution.

Taken together our data demonstrate that inhibition of the CXCL12/CXCR4 signaling axis drives the resolution of inflammation by increasing neutrophil reverse migration, and supports the hypothesis that neutrophil desensitization to gradients at the wound site results in their reverse migration away from the wound site (16, 18). These data add to the existing evidence that neutrophil reverse migration can be targeted pharmacologically to drive the resolution of inflammation.

## Data Availability

The datasets analyzed for this study can be found in the Blood Atlas of Single Cells in zebrafish (BASiCz) database (https://www.sanger.ac.uk/science/tools/basicz) and a database of single cell RNA sequencing of larval zebrafish neutrophils, data deposited on GEO under accession number GSE78954 (https://www.ncbi.nlm.nih.gov/geo/query/acc.cgi?acc=GSE78954).

## Ethics Statement

The animal study was reviewed and approved by UK Home Office approved facilities at The Bateson Centre aquaria at the University of Sheffield.

## Author Contributions

HI performed all experiments with assistance from CL, AR, KH, and PE. HI and KH analyzed data. SR and PE conceived the study and designed experiments. SR, PE, and LP provided scientific expert knowledge. HI wrote the manuscript with significant input from all authors.

### Conflict of Interest Statement

The authors declare that the research was conducted in the absence of any commercial or financial relationships that could be construed as a potential conflict of interest.
