# Supplementary material for: The CXCL12/CXCR4 Signaling Axis Retains Neutrophils at Inflammatory Sites in Zebrafish
Source: Front Immunol. 2019 Jul 31;10:1784. doi: 10.3389/fimmu.2019.01784 (PMC6684839; doi:10.3389/fimmu.2019.01784)
Supplement: Supplementary file 1 [file Data_Sheet_1.PDF]

Supplementary Material

Supplementary Figures

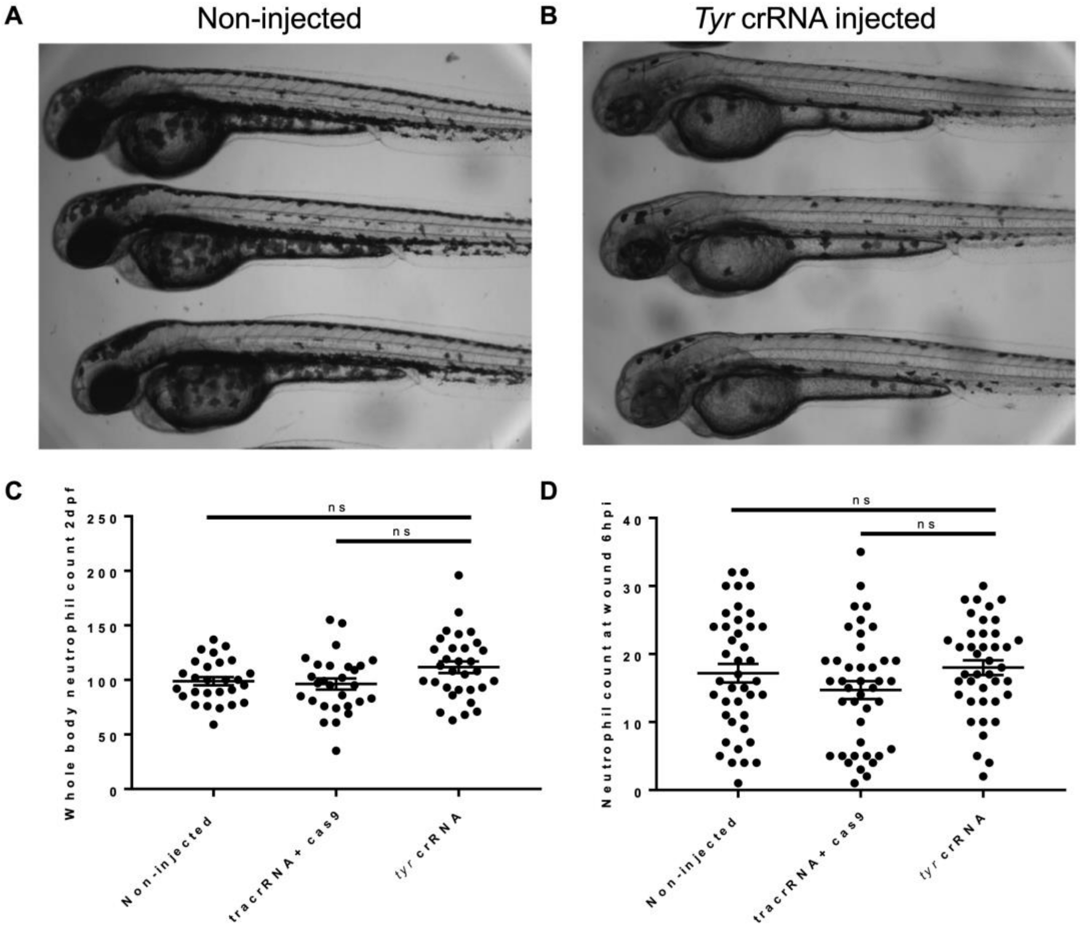

Supplemental Figure 1. CRISPR/Cas9 knockdown of *tyrosinase* does not affect neutrophil function

A-B. Representative images of 2dpf mpx:GFP non-injected (**A**) and *tyrosinase* (**B**) mosaic pigment phenotypes. **C.** Whole body neutrophil counts in non-injected, vehicle control tracrRNA + cas9 protein injected and *tyrosinase* crRNA injected larvae. **D.** Neutrophils recruited to the injury site at 6hpi in 2dpf non-injected, vehicle control tracrRNA + cas9 protein injected and *tyrosinase* crRNA injected larvae. *(Error bars shown are mean ± SEM. Groups were analysed using an ordinary one-way ANOVA and adjusted using Tukeys multi comparison test, n=30 from 3 independent repeats*).

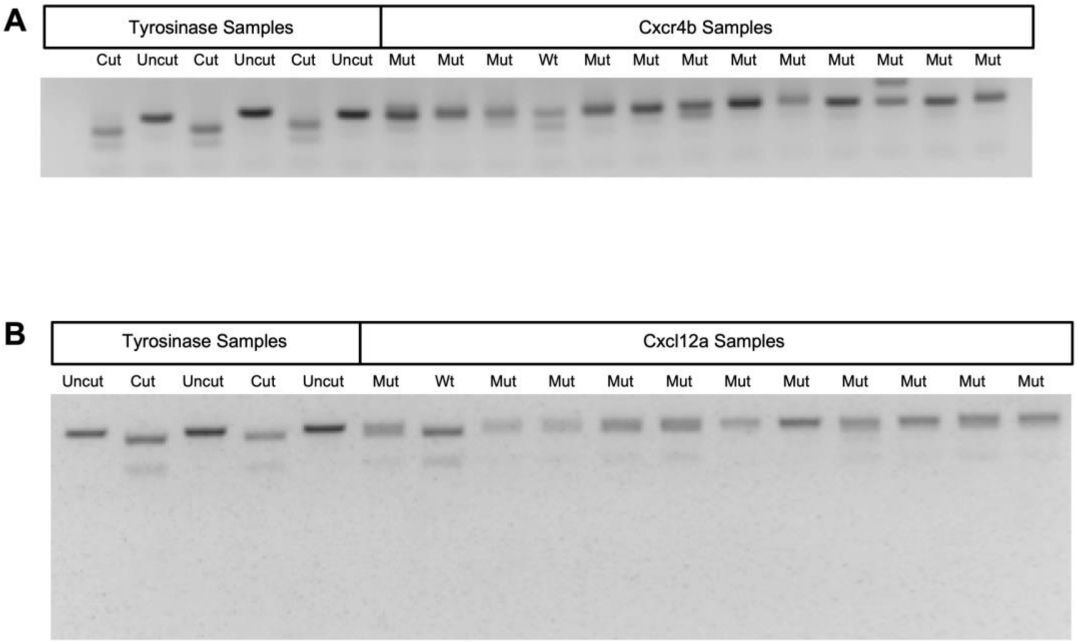

Supplemental Figure 2. Genotyping of *cxcr4b* and *cxcl12a* CRISPR knockdown using restriction digest

A Electrophoresis gel for *cxcr4b* crispants at 2dpf. Lanes 1-6 Control *Tyr* injected larvae. Lanes 1,3,5 PCR produced incubated with bsII restriction enzyme, lanes 2,4,6 undigested PCR product. Lanes 7-19 *cxcr4b* crRNA injected larvae where PCR product has been digested using bsII. **B** Electrophoresis gel for *cxcl12a* crispants at 2dpf. Lanes 1-5 Control *Tyr* injected larvae. Lanes 1,3,5 Undigested PCR product, lanes 2,4 PCR produced incubated with bstXi restriction enzyme. Lanes 6-18 *cxcl12a* crRNA injected larvae where PCR product has been digested using bstXi.
